# Association between a genetic variant in the serotonin transporter gene (*SLC6A4*) and suicidal behavior in patients with schizophrenia

**DOI:** 10.1186/1744-9081-8-24

**Published:** 2012-05-17

**Authors:** Eva Lindholm Carlström, Peter Saetre, Anders Rosengren, Johan H Thygesen, Srdjan Djurovic, Ingrid Melle, Ole A Andreassen, Thomas Werge, Ingrid Agartz, Håkan Hall, Lars Terenius, Erik G Jönsson

**Affiliations:** 1 Department of Clinical Neuroscience, HUBIN Project, Karolinska Institutet and Hospital, R5:00, Stockholm, Sweden; 2 Research Institute of Biological Psychiatry, Copenhagen University Hospital, Mental Health Centre Sct. Hans, Roskilde, Denmark; 3 TOP project, Division of Mental Health and Addiction, Oslo University Hospital & Institute of Clinical Medicine, University of Oslo, Oslo, Norway; 4 Institute of Clinical Medicine, University of Oslo, Psychiatry Section, Vinderen, Oslo, Norway; 5 Department of Psychiatric Research, Diakonhjemmet Hospital, Oslo, Norway

**Keywords:** Suicide ideation, Serotonin transporter gene, Association, Schizophrenia

## Abstract

**Background:**

The serotonin (5-hydroxytryptamin; 5-HT) system has a central role in the circuitry of cognition and emotions. Multiple lines of evidence suggest that genetic variation in the serotonin transporter gene (*SLC6A4*; *5-HTT*) is associated with schizophrenia and suicidal behavior. In this study, we wanted to elucidate whether *SLC6A4* variations is involved in attempted suicide among patients with schizophrenia in a Scandinavian case–control sample.

**Methods:**

Patients diagnosed with schizophrenia from three Scandinavian samples were assessed for presence or absence of suicide attempts, based on record reviews and interview data. Seven *SLC6A4* single nucleotide polymorphisms (SNPs) were genotyped in 837 schizophrenia patients and 1,473 control individuals. Association analyses and statistical evaluations were performed with the program UNPHASED (version 3.0.9).

**Results:**

We observed an allele association between the SNP rs16965628, located in intron one of *SLC6A4*, and attempted suicide (adjusted p-value 0.01), among patients with schizophrenia. No association was found to a diagnosis of schizophrenia, when patients were compared to healthy control individuals.

**Conclusion:**

The gene *SLC6A4* appears to be involved in suicidal ideation among patients with schizophrenia. Independent replication is needed before more firm conclusions can be drawn.

## Background

The lifetime risk of committing suicide among patients with schizophrenia is approximately 5% [1,2]. Also attempted suicide is common, with estimates ranging from 20% to 40% [3]. The heritable component of suicide attempt is partly related to psychiatric disorders but also partly independent of them [4-6].

The serotonin (5-hydroxytryptamin; 5-HT) system has a central role in the circuitry of cognition and emotions and exerts significant effects on anxiety, mood, impulsivity, sleep, ingestive behavior, reward systems, and psychosis [7]. Pharmacological agents targeting central 5-HT system has substantial effects on emotional behaviors [8-10]. Moreover, several reports imply that 5-HT may be involved in pathophysiological events associated with suicide [11-15]. In fact, postmortem examination of suicide victims shows significantly lower serotonin transporter binding in the prefrontal cortex [16].

Epidemiological and genetic studies indicate that there is a genetic component to suicidal behavior [17]. For example, a single nucleotide polymorphism (SNP) (rs1800532, A218C) in the tryptophan hydroxylase 1 gene (*TPH1*), which encodes a rate-limiting enzyme involved in the development of 5-HT, was previously found to be associated with suicide or suicidal attempt [18,19]. The same SNP was also investigated by us in the Scandinavian case–control material used in the present study [20]. However, no association was detected between *TPH1* A218C and suicide attempt among the patients, although an association was found to schizophrenia.

**Figure 1:**
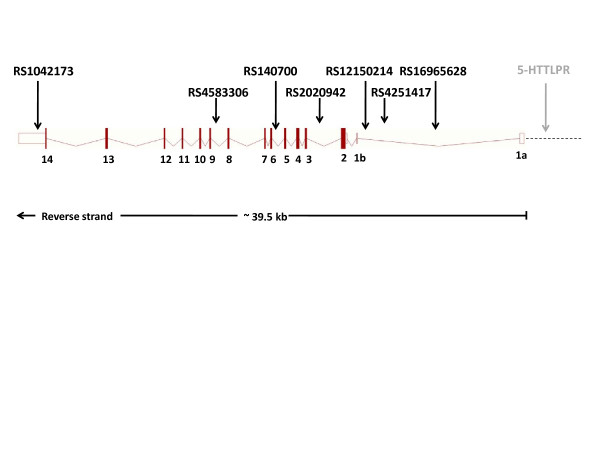
**Genomic structure of the *SLC6A4 *gene.** Exons (1a–14) are represented by filled boxes and untranslated regions are represented with open boxes. The 5’-UTR is indicated with a dashed line. The SNPs in the figure was included in present study. The 5-HTTLPR locus marked with grey color, was not included in the present association study.

Another gene in the serotonin system, reported several times to be involved in the risk of attempting suicide, is the serotonin transporter gene (*SLC6A4* or *5-HTT*) [11,12,19,21]. The human *SLC6A4* gene (Figure 1), located on chromosome 17q11.2, is about 39,500 base pairs long and contains 14 exons, of which exons 1a and 1b are alternatively transcribed. The gene encodes a membrane protein that plays a major role in the regulation of synaptic serotonin concentrations by recycling serotonin from the synaptic cleft.

Results from studies of 5-HTT knockout mouse have provided important clues regarding downstream effects of altered 5-HT function on behavior and the development of the brain, such as regulation of cell proliferation, migration and differentiation of neuronal tissue [22,23]. Interestingly, early alterations in 5-HT availability during the development of the 5-HT system might impact the distribution and density of specific 5-HT receptors, thereby altering the postsynaptic effects of 5-HT on target neurons and the reactivity of these neurons to specific stimuli [24].

In recent years, five genome-wide studies analyzing suicidal behavior in patients with bipolar disorder, alcoholism and major depression identified candidate regions on chromosome 2p11-12, 2p25 and 6q25-26 [6,25-29]. As far as we know, no genes associated with suicidal behavior have been reported in these regions yet. Neither are we aware of any genome wide studies analyzing suicidal behavior in patients with schizophrenia.

In the present study, we wanted to further explore the possible involvement of the serotonin system in suicide among patients with schizophrenia, by analyzing genetic variations in the *SLC6A4* gene using a Scandinavian case–control sample. Scandinavian countries are generally considered to be well suited for genetic studies, as the populations are ethnically homogeneous and only recently have been subject to non-Caucasian immigration. Seven SNPs located in the *SLC6A4* gene (Figure 1), were selected and genotyped in 837 patients suffering from schizophrenia and related disorders, of which 738 had information on suicidal behavior, and in 1,473 control individuals.

## Methods

### Clinical samples

The clinical samples originate from the Scandinavian Collaboration on Psychiatric Etiology (SCOPE) and were collected in Denmark (DK), Norway (NO), and Sweden (SE). Affected individuals were diagnosed according to ICD-10 (DK) or DSM-III-R/DSM-IV (NO and SE). All individuals were born in Scandinavia and of Caucasian origin, and the vast majority had two Scandinavian born parents. Detailed description of the samples was reported earlier [20,30-33]. The total sample set included 837 patients, of which 734 were diagnosed with schizophrenia, 87 with schizoaffective disorder and 16 with schizophreniform disorder, and 1473 control individuals (Table 1). Patients (N = 738) were assessed for presence or absence of suicide attempts based on record reviews (DK, SE) and interview data (NO, SE). Any previous self-harm in combination with suicidal ideation, documented or reported, was regarded as a suicide attempt. For the present analyses, subjects were divided into those who had made at least one suicide attempt and those who had not attempted suicide. The Danish Scientific Committees, the Danish Data Protection Agency, the Norwegian Scientific-Ethical Committees, the Norwegian Data Protection Agency, the Ethical Committee of the Karolinska Hospital, the Stockholm Regional Ethical Committee and the Swedish Data Inspection Board all approved the study. All participants had given informed consent prior to inclusion in the study.

**Table 1:** **Characteristics of Danish (DK), Norwegian (NO), and Swedish (SE) samples analyzed for association between serotonin transporter gene (*SLC6A4 *) polymorphisms and schizophrenia and suicide attempt among affected (cases) and control individuals**

**Country**	**Characteristics**	**Cases**	**Controls**
DK	N	420	1004
	Gender (% women)	42.6	41.5
	Age	43.9 ± 12.3	43.2 ± 11.8
	Age of onset	27.2 ± 8.9	
	No of suicide attempters	153	
NO	N	162	177
	Gender (% women)	46.3	55.5
	Age	37.2 ± 10.7	38.7 ± 10.3
	Age of onset	27.6 ± 8.7	
	No of suicide attempters	42	
SE	N	255	292
	Gender (% women)	37.2	37.7
	Age	54.1 ± 15.2	50.3 ± 10.1
	Age of onset	24.6 ± 6.9	
	No of suicide attempters	95	

### SNP Genotyping

Seven SNPs in the *SLC6A4* gene, previously reported to be associated with schizophrenia, were analyzed. Genomic DNA was extracted from whole blood samples, and the polymorphisms were genotyped at the SNP Technology Platform in Uppsala, Sweden (http://www.genotyping.se), using the Illumina BeadStation 500GX and the 1536-plex Illumina Golden Gate assay (Illumina, Inc., San Diego, CA). The sample success rate was on average 99.7% for the genotyped SNPs and the reproducibility of the genotyping was 100% according to duplicate analysis of 2.6% of the genotypes.

### Statistical analyses

Hardy–Weinberg (HW) equilibrium was tested in the control samples using Fisher’s exact test as implemented in PEDSTATS [34]. Accounting for the number of tested markers, no SNP deviated significantly from HW equilibrium. Linkage disequlibrium (D’ and R^2^) between SNP pairs, and haplotype block structure [35] were determined with Haploview 4.0 [36] (Additional file 1: Figure S1). The fixation index (FST) was calculated for each SNP separately, and all loci combined, in the control sample with FSTAT, grouping controls by country of origin. No evidence of population stratification was evident from the data: the fixation index for all combined loci was 0.003 (0.001-0.005) (95% bootstrap confidence interval).

Association analysis comparing allele and genotype frequencies between suicide attempters and non-attempters within the patient group and between affected individuals and controls were performed with UNPHASED (version 3.0.9) [37,38]. To account for possible population stratification and the effect of sex, we included country of origin and sex as confounding factors in the analyses. To examine whether genetic associations were homogenous, we carried out separate analyses testing if country and gender modified the association. Correction for multiple testing was done by permutation tests (n = 1000).

## Results

Out of the seven markers tested, only rs16965628 located in the *SLC6A4* gene, showed significant association to suicide attempt (P-value for allele association = 0.00092) and the result remained significant also after correction for multiple testing (P-value = 0.01) (Table 2). The minor allele C was underrepresented among patients with records of suicidal behavior.

**Table 2:** **Serotonin transporter (*SLC6A4 *) single nucleotide polymorphisms (SNPs) investigated in suicidal attempters (SA) and non-suicidal (No SA) attempters with a schizophrenia diagnosis**

**SNP**	**SNP localization**	**Position**		**MAF**	**Base**	**Genotype counts**			**Allele association**		**Genotype association**		
						**(SA/No SA)**				**OR**		**OR**	**OR**
			**(no of bases)**		**(1/2)**	**1 1**	**1 2**	**2 2**	**P-value**	**2 vs 1**	**P-value**	**12 vs 11**	**22 vs 11**
Rs1042173	3'-UTR	28525011		0.47	A/C	70/125	144/195	75/104	0.218	1.14	0.27	1.34	1.29
Rs4583306	Intron 8	28538715	13704	0.03	A/G	77/134	150/201	60/88	0.335	1.11	0.29	1.33	1.20
Rs140700	Intron 5	28543389	4674	0.08	C/T	243/348	43/79	2/0	0.560	0.89	0.11	0.80	2.26E9
Rs2020942	Intron 2	28546914	3525	0.39	C/T	108/157	135/201	44/68	0.777	0.97	0.94	0.99	0.93
Rs12150214	Intron 1	28550888	3974	0.17	G/C	213/301	67/117	10/8	0.735	0.95	0.23	0.81	1.72
Rs4251417	Intron 1	28551858	970	0.10	C/T	227/347	55/77	4/1	0.338	1.19	0.18	1.07	6.26
Rs16965628	Intron 1	28555425	3567	0.05	G/C	272/374	15/51	0/1	0.0009 (0.01)	0.39	0.004 (0.02)	0.40	3.92E-5

The table shows nominal allele and genotype association results with gender and country as covariates.

Chromosomal position, minor allele frequency (MAF), SNP-alleles (Base), genotype counts, P-values and odds ratios (OR) for allele and genotype association for the seven SNP markers are presented in the table. Global significant P-values are shown in parenthesis.

We also performed genotype association analyses. However, due to the low frequency of the minor allele of rs16965628 (0.05) only one individual was CC homozygous, and consequently the same association signal was captured with the allele and the genotype test. We did not find any evidence of heterogeneity in the association signal between the three countries, nor between male and female patients for this marker (P-value = 0.75 and 0.17, for country and gender as modifiers, respectively). In other words, all countries and both genders contributed to the observed association between rs16965628 and suicidal behavior.

For completeness, the allele and genotype frequencies of the seven polymorphisms in patients with suicidal behavior were also contrasted against the frequencies in healthy controls. The results were similar to the comparison within the patient group, and the rs16965628 C-allele was significantly lower among individuals with suicidal behavior than in the controls (p-value = 0.01 after correction for multiple testing, data not shown).

Since genetic variants in the *SLC6A4* were previously found to be associated with schizophrenia in other sample sets [39-44], we analyzed the association between the seven SNPs and schizophrenia in the Scandinavian case–control sample. Two SNPs were weakly associated with the disease (Table 3), but after correction for multiple testing no association was statistically significant (P-value > 0.10).

**Table 3:** **Serotonin transporter (*SLC6A4 *) single nucleotide polymorphisms (SNPs) investigated in patients with schizophrenia and control subjects**

**SNP**	**Base**	**MAF**	**Genotype counts (schizophrenia/control)**			**Allele association**			**Genotype association**			
			**Allele 1**		**Allele 2**		**OR**				**OR**	**OR**
	**(1/2)**		**Homozyogotes**	**Heterozygotes**	**Homozygotes**	**p-value**	**2 vs 1**		**p-value**		**12 vs 11**	**22 vs 11**
Rs1042173	A/C	0.46	223/410	402/770	207/291	0.083		1.11	0.022		0.93	1.27
Rs4583306	A/G	0.42	276/475	410/755	173/237	0.020		1.16	0.020		1.04	1.40
Rs140700	C/T	0.09	689/1233	140/221	2/10	0.435		1.09	0.171		1.17	0.39
Rs2020942	C/T	0.14	313/528	390/714	128/228	0.359		0.94	0.518		0.90	0.91
Rs12150214	G/C	0.18	595/982	219/438	21/46	0.123		0.88	0.282		0.86	0.83
Rs4251417	C/T	0.09	673/1204	148/257	9/7	0.214		1.14	0.123		1.07	2.78
Rs16965628	G/C	0.06	753/1310	77/152	1/9	0.192		0.83	0.125		0.91	0.19

The table shows nominal allele and genotype association results with gender and country as covariates.

Nominal allele and genotype association results are reported with gender and country as covariates. MAF, minor allele frequencies in control individuals (allele 2 in the table).

Nominally significant; after correction for multiple testing using permutation test, all P- values > 0.10.

We analyzed linkage disequilibrium (LD) between the polymorphisms and found two blocks. All markers, except rs16965628, were located in the observed LD blocks (Additional file 1: Figure S1). Haplotype association analyzes for the two blocks, with respect to suicidal attempt and schizophrenia susceptibility, were performed with UNPHASED. The haplotype and single marker analyzes gave similar results, i.e. there were no significant haplotype association with neither phenotype (p > 0.106, data not shown).

## Discussion

In the present study we found an association between the *SLC6A4* rs16965628 polymorphism and attempted suicide among patients with schizophrenia (P-value = 0.00092 and global P-value = 0.01). The odds ratio was 0.39, indicating that the presence of the minor C-allele protects against suicidal behavior. The rs16965628 polymorphism is located in the first intron of the *SLC6A4* gene, approximately 9 kb downstream of promoter region. The most studied polymorphisms in the *SLC6A4* gene are a 44-base pair insertion–deletion in the promoter region, generating the major L (LA and LG) and S-alleles, and a 17-bp variable number of tandem repeats (VNTR) in the second intron [45]. During the years conflicting results have been reported regarding genetic variants in the *SLC6A4* gene and suicidal ideation [46]. The ambiguous results from different studies possibly reflect insufficient sample sizes to obtain adequate statistical power, and heterogeneity between populations [47]. However, the two latest of three meta-analyses report association between *SLC6A4* variants and suicidal behavior [11,12,21]. In the latest and largest meta-analysis, including 39 different association studies (covering all published studies up to 2006), a significant association was found between 5-HTTLPR and suicide attempts/suicide. The L-allele was underrepresented among individuals with suicidal behavior (odds ratio was 0.88), suggesting that the investigated gene variant had a protective effect against suicide [11].

The mechanism behind this association is unknown, but the 5-HTTLPR polymorphism appears to affect *SLC6A4* gene expression. That is, the transcription of the *SLC6A4* gene is lower in the presence of the S-allele as compared to the L or the LA-alleles [48,49]. As mentioned in the introduction, postmortem examination of suicide victims shows significantly lower serotonin transporter binding in the prefrontal cortex [16]. Thus it is possible that the protective effect of the L-allele is the result of elevated expression of the serotonin transporter protein, leading to an overall increase in serotonin transporter binding.

The relative abundance of allele transcripts of the serotonin transporter gene in human cell lines from HapMap CEPH trios, confirm the effects of 5-HTTLPR variation on the transcription of the *SLC6A4* gene [50]. However, in these cell lines variation in the rs16965628 SNP was associated with considerably more transcriptional variation than the 5-HTTLPR polymorphism, and the rs16965628 C-allele was linked with increased transcription (the mean allele G/C transcript ratio was 0.47) [50]. Thus we speculate that the decreased frequency of suicidal behavior among schizophrenic patients that carried the rs16965628 C-allele, observed in this study, may have been caused by increased transcription of the *SLC6A4 gene* and a corresponding increase in serotonin transporter binding.

The gene *SLC6A4* has frequently been implicated in psychiatric disorders [51]. Meta-analyses have reported association between 5-HTTLPR S-variant - and sometimes also the 17-bp VNTR in intron 2 polymorphism - and bipolar disorder [52-55], co-morbid bipolar disorder and tobacco use disorder [56], alcohol dependence [57,58], major depression [59,60], antidepressant-induced mania [61,62], a modulation effect of 5-HTTLPR on stress and depression [63], however highly discussed [64], and anxiety-related traits [65-67], almost all disorders with elevated rates of suicide or suicide attempts. Almost all of the above mentioned disorders show elevated rates of suicide or suicide attempts. In schizophrenia, the 5-HTTLPR and 17-bp VNTR in intron 2 polymorphisms have been associated with the disorder in individual studies and the 17-bp VNTR also in several meta-analyses [68-70]. However, the latest update in the SzGene database (http://www.szgene.org) indicates lack of overall allele association. In previous studies, 5-HTTLPR and schizophrenia was investigated by us using Swedish case–control samples, partly overlapping with the present. The results indicated that alleles within the gene were associated with age of onset [71] and disease [44]. Other researchers reported that a haplotype, including markers 5-HTTLPR and rs16965628, was associated with obsessive compulsive disorder [72]. Furthermore, rs16965628 was found to modulate task-related activation in ventral prefrontal cortex in patients with posttraumatic stress disorder [73]. Patients with obsessive compulsive disorder and posttraumatic stress disorder are known to have an elevated risk of committing suicide [74,75].

In the present study, we did not find any significant association between genetic variants in *SLC6A4* and schizophrenia although an association was found to suicide attempt. This may seem contradictive but previous epidemiological studies including monozygotic and dizygotic twins showed an elevated risk of attempting suicide even after controlling for psychiatric disorders, indicating that genetic factors independent of psychiatric disorders affect the risk of suicide attempt [4,5]. Thus our results do not support that the investigated SNPs are associated with a substantial increase in the disease risk in the Scandinavian population. However, even though our sample size was large, it does not have the power to unambiguously detect weak signals, and thus a true association with an odds ratio below 1.2 cannot be ruled out [76].

## Conclusions

The present results provide support for an association between a relatively uncommon *SLC6A4* polymorphism and suicidal behavior among schizophrenic patients of Scandinavian origin. As the marker was not associated with the disease, the decreased risk for suicidal behavior appears to be unlinked to that of schizophrenia susceptibility. Although the estimated effect of the rs1695628 C-allele was substantial, its low frequency suggests that the potential contribution to variation in suicidal behavior in the population would be limited. Further studies in independent samples are needed to establish a link between rs1695628 and suicidal behavior in the Scandinavian and other populations.

## Abbreviations

SNP: Single nucleotide polymorphism; *SLC6A4* (5-*HTT*): Serotonin transporter gene; TPH1: Tryptophan hydroxylase 1; SCOPE: Scandinavian Collaboration on Psychiatric Etiology; DK: Denmark; NO: Norway; SE: Sweden; ICD: International Statistical Classification of Diseases and Related Health Problems; DSM: Diagnostic and Statistical Manual of Mental Disorders; DNA: Deoxyribonucleic acid; LD: Linkage Disequilibrium.

## Competing interests

The authors declare that they have no competing interests.

## Authors’ contributions

ELC wrote the draft and coordinated the preparation of the manuscript. PS participated in the study design, performed the statistical analyses and was involved in the manuscript preparation. AR and JHT contributed with data collection (DK sample). SD participated in the study design and contributed with data collection (NO sample). IM participated in the study design, clinical characterization and contributed with data collection (NO sample). OAA participated in the study design and contributed with data collection (NO sample). TW participated in the study design and contributed with data collection (DK sample). IA, HH and LT participated in the study design and contributed with data collection (SE sample). EGJ participated in the study design, clinical characterization, contributed with data collection (SE sample) and was involved in the manuscript preparation. All authors contributed to and have approved the final manuscript.

## Supplementary Material

Additional file 1 **Figure S1.** Linkage disequilibrium (LD) structure in the three Scandinavian control samples of seven serotonin transporter gene (*SLC6A4*) single nucleotide polymorphisms. Haplotype block structure (outlined), D’ (numbers, 100 not printed) and r^2^ (shadings) are given. The figure shows pair-wise LD among the seven *SLC6A4* SNPs, as calculated by the Haploview 4.0 software. High r^2^ values with strong linkage disequilibrium are indicated by dark color. Gray and white colors indicate weak linkages with low r^2^ values. The genetic positions of the SNPs in the *SLC6A4* gene are indicated in the white bar above the LD-plot. Click here for file
