# Supplementary material for: Association between a genetic variant in the serotonin transporter gene (SLC6A4) and suicidal behavior in patients with schizophrenia
Source: Behav Brain Funct. 2012 May 17;8:24. doi: 10.1186/1744-9081-8-24 (PMC3527134; doi:10.1186/1744-9081-8-24)
Supplement: Additional file 1 — Figure S1. Linkage disequilibrium (LD) structure in the three Scandinavian control samples of seven serotonin transporter gene (SLC6A4) single nucleotide polymorphisms. Haplotype block structure (outlined), D’ (numbers, 100 not printed) and r2 (shadings) are given. The figure shows pair-wise LD among the seven SLC6A4 SNPs, as calculated by the Haploview 4.0 software. High r2 values with strong linkage disequilibrium are indicated by dark color. Gray and white colors indicate weak linkages with low r2 values. The genetic positions of the SNPs in the SLC6A4 gene are indicated in the white bar above the LD-plot. [file 1744-9081-8-24-S1.pdf]

Figure S1. Linkage disequilibrium (LD) structure in the three Scandinavian control samples of seven serotonin transporter gene (*SLC6A4*) single nucleotide polymorphisms.

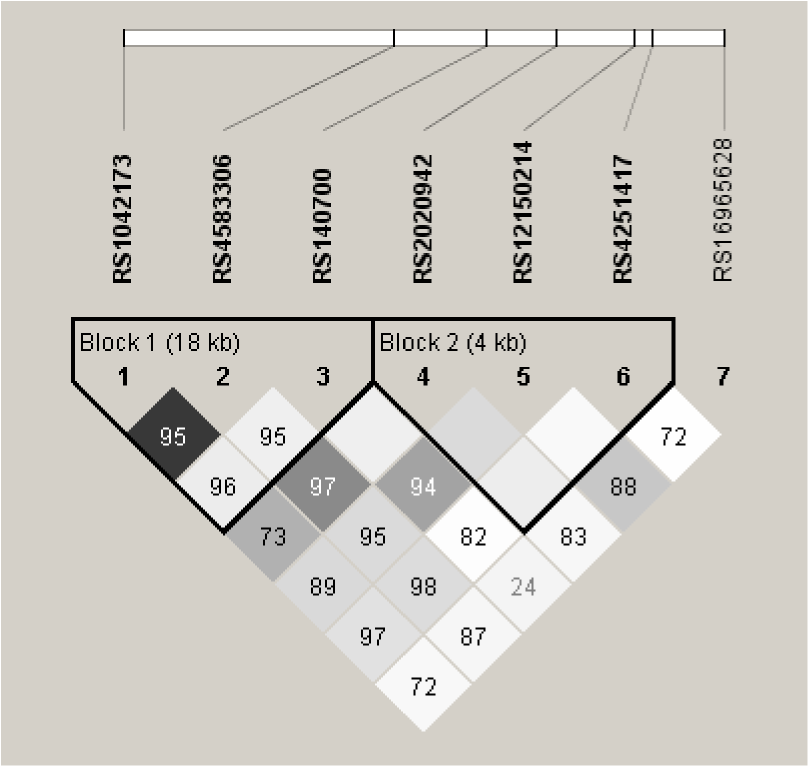

The figure shows pair-wise LD among the seven *SLC6A4* SNPs, as calculated by the Haploview 4.0 software. High D′ values with strong linkage disequilibrium are indicated by dark color. Gray and white colors indicate weak linkages with low D′ values. The genetic positions of the SNPs in the SLC6A4 gene are indicated in the white bar above the LD-plot.
